# Does the sixth wave of COVID‐19 break in Okinawa?

**DOI:** 10.1002/jgf2.550

**Published:** 2022-05-02

**Authors:** Masaki Tomochi, Mitsuo Kono

**Affiliations:** ^1^ Department of Economics, Okinawa International University Okinawa Japan; ^2^ Faculty of Policy Studies Chuo University Tokyo Japan

**Keywords:** COVID‐19, forecast, infectious diseases, vaccination policy, VSIIR model

## Abstract

**Background:**

We aimed to forecast possible situations of the COVID‐19 spreading for Okinawa Prefecture in Japan.

**Methods:**

The VSIIR model is proposed to extend the SIIR model to include vaccine effects where the parameter v denotes the vaccination rate and is treated as a control parameter on which possible situations for Okinawa would depend.

**Results:**

It is shown that the infection ends without spreading if $v>d_{1}+d_{2}$ is satisfied where $1/d_{i}$ refers to the antibody duration, i=1 for infection and i=2 for vaccination, respectively.

**Conclusion:**

It is important to set a vaccination policy that can save lives and maintain daily life at the same time.

## Graphical abstract

The VSIIR model that describes the spread of infection by taking account of the characteristics of COVID‐19 is proposed to extend the SIIR model to include vaccine effects, aiming to forecast possible situations for Okinawa Prefecture in Japan at the end of its fifth wave of infection.

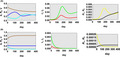

## 1 VSIIR MODEL

The SIIR model for COVID‐19 spreading has shown to reproduce the observed data of those being positive to the PCR test and clarify that one of the basic features of COVID‐19 is asymptomatic individuals who are mostly left unchecked and responsible for the spread of infection.^1^, ^2^ In this paper, the VSIIR model is proposed to extend the SIIR model to include vaccine effects, aiming to forecast possible situations for Okinawa Prefecture in Japan at the end of its fifth wave of infection.

Here, variables in VSIIR model at time t are $S\left(t\right)$ as susceptible population, $I_{1}\left(t\right)$ as presymptomatic population (infectious), $I_{2}\left(t\right)$ as asymptomatic population (infectious), $R_{1}\left(t\right)$ as symptomatic population (not infectious due to quarantine), $R_{2}\left(t\right)$ as recovered population (with antibody and not infectious), $R_{3}\left(t\right)$ as fatalities by COVID‐19 (not infectious), and $V\left(t\right)$ as vaccinated population (with antibody and not infectious). Then, the interrelationship among the above variables is described by the following coupled differential equations from (1) to (7):

(1) $$\frac{dS\left(t\right)}{dt}=-\mathrm{\beta S}\left(t\right)\left(I_{1}\left(t\right)+I_{2}\left(t\right)\right)+d_{1}R_{2}\left(t\right)+d_{2}V\left(t\right)-\mathrm{vS}\left(t\right),$$

(2) $$\frac{dI_{1}\left(t\right)}{dt}=\mathrm{\beta S}\left(t\right)\left(I_{1}\left(t\right)+I_{2}\left(t\right)\right)-\left(b_{1}+b_{2}+v\right)I_{1}\left(t\right),$$

(3) $$\frac{dI_{2}\left(t\right)}{dt}=b_{2}I_{1}\left(t\right)-\left(c_{1}+v\right)I_{2}\left(t\right),$$

(4) $$\frac{dR_{1}\left(t\right)}{dt}=b_{1}I_{1}\left(t\right)-\left(c_{2}+c_{3}\right)R_{1}\left(t\right),$$

(4) $$\frac{dR_{2}\left(t\right)}{dt}=c_{1}I_{2}\left(t\right)+c_{2}R_{2}\left(t\right)-\left(d_{1}+v\right)R_{2}\left(t\right),$$

(6) $$\frac{dR_{3}\left(t\right)}{dt}=c_{3}R_{1}\left(t\right),$$

(7) $$\frac{dV\left(t\right)}{dt}=v\left(S\left(t\right)+I_{1}\left(t\right)+I_{2}\left(t\right)+R_{2}\left(t\right)\right)-d_{2}V\left(t\right).$$

In the VSIIR model, the vaccination rate is given as v. Usually, vaccines are supposed to be given to those who are uninfected (non‐antibody) and ex‐infected (antibody‐deficient); however, since it would be realistic to inoculate individuals randomly except those who are symptomatic and quarantined, not only the susceptible population $S\left(t\right)$ but also presymptomatic population $I_{1}\left(t\right)$, asymptomatic population $I_{2}\left(t\right)$, and recovered population $R_{2}\left(t\right)$ are treated as subjects in need of vaccination in the model. For this reason, there will be cases where antibody acquisition is duplicated. In fact, the cost of distinguishing uninfected individuals from asymptomatic individuals would be enormous and random vaccination is thought to be more economical and practical in procedure. Here, the antibody duration of the infected and recovered individuals ($1/d_{1}$) and that of those who are vaccinated ($1/d_{2}$) are set as finite. Note that a conservation law exists as well in the VSIIR model and is given as

$$N=S\left(t\right)+I_{1}\left(t\right)+I_{2}\left(t\right)+R_{1}\left(t\right)+R_{2}\left(t\right)+R_{3}\left(t\right)+V\left(t\right).$$

## 2 PARAMETERS AND INITIAL VALUES

The parameters of the VSIIR model are determined so that the data of the number of new positives of COVID‐19 in Okinawa up to the fifth wave match the value of $R_{1}\left(t\right)$ in the SIIR model:

$$t_{1}=5,\;t_{2}=17,$$

(10) $$\beta =0.2,\;b_{1}=0.06/t_{1},\;b_{2}=1/t_{1}-b_{1},\;c_{1}=1/t_{2},\;c_{2}=0.942/t_{2},\;c_{3}=1/t_{2}-c_{2}$$

where $t_{1}$ and $t_{2}$ denote the incubation period and the period of the onset, respectively.^3^, ^4^, ^5^ For simplicity, regardless of the antibody acquisition route, the duration of the antibody is assumed to be the same for both the recovery from infection and the vaccine, and

(111) $$d_{1}=d_{2}=0.005.$$

is set since there are several findings that the duration of the antibody is about 6 months.^6^ In the following section, the parameter v is treated as a control parameter on which the sixth wave of infection in Okinawa would depend.

The initial value of the VSIIR model after the fifth wave is given as

(11) $$S\left(0\right)=\left(1-q_{2}\right)a_{2}+\left(1-q_{4}\right)a_{4}+\left(1-q_{5}\right)a_{5},\;I_{1}\left(0\right)=I_{10},\;I_{2}\left(0\right)=I_{20},$$

(12) $$R_{1}\left(0\right)=R_{10},\;R_{2}\left(0\right)=\left(1-q_{1}\right)a_{1}+\left(1-q_{3}\right)a_{3},\;R_{3}\left(0\right)=0,\;V\left(0\right)=w_{1}$$

with the values shown in Table 1 which are obtained from officially reported data of COVID‐19 in Okinawa and the asymptomatic individuals based on the SIIR model. Note that the following constraint condition(13)
$$w_{1}=\sum_{i=1}^{5}q_{i}a_{i}\to 1=\sum_{i=1}^{5}q_{i}a_{i}/w_{1}=0.04q_{1}+0.01q_{2}+0.63q_{3}+0.23q_{4}+0.70q_{5}\;$$
implies $q_{i}$ cannot be freely chosen.

**TABLE 1:** COVID‐19 status after the fifth wave (October 25, 2021) in Okinawa

Population of Okinawa (N=1,485,195)	Antibody	Cumulative number of individuals (rate)	Vaccination rate	Nonvaccination rate
Recovered symptomatic individuals	Yes	$a_{1}=36,794$ (2.48%)	$q_{1}$	$1-q_{1}$
	No	$a_{2}=13,373$ (0.90%)	$q_{2}$	$1-q_{2}$
Recovered asymptomatic individuals	Yes	$a_{3}=577,666$ (38.89%)	$q_{3}$	$1-q_{3}$
	No	$a_{4}=209,956$ (14.14%)	$q_{4}$	$1-q_{4}$
Individuals not infected yet		$a_{5}=641,881$ (43.22%)	$q_{5}$	$1-q_{5}$
Infectious individuals $I_{10}+I_{20}=5267\left(0.35\%\right)$	Presymptomatic	$I_{10}=290$ (0.02%)	0	1
	Asymptomatic	$I_{20}=4,977$ (0.33%)	0	1
Symptomatic individuals		$R_{10}=258$ (0.02%)	0	1
Total		N=1,485,195 (100%)	$w_{1}=911,464$ (61.37%)	$w_{0}=573,731$ (38.63%)

## 3 SIMULATION RESULTS

In the following simulation, $q_{i}$ is set as uniformly equal probability, namely $q_{1}=q_{2}=q_{3}=q_{4}=q_{5}=w_{1}/\sum_{i=1}^{5}a_{i}\approx 0.62$, so as to satisfy Equation (8). This is because random vaccination is thought to be more economical and practical. At this point, uninfected individuals and recovered asymptomatic individuals who lost antibodies will account for the major targets for vaccination, so the initial susceptible population is small, 22% of the total ($\approx S\left(0\right)/N$), and the effective reproduction number is calculated as 0.939 ($\approx \beta S\left(0\right)t_{1}\left(1+b_{2}t_{2}\right)$).^7^

**FIGURE 1:**
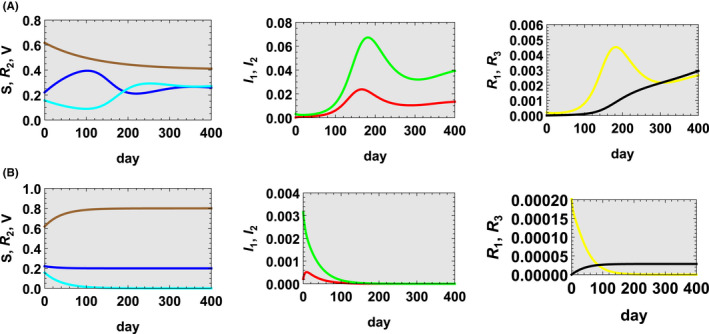
(A) Case 1 (v=0.00341) and (B) case 2 (v=0.02). $S\left(t\right)$ in blue, $I_{1}\left(t\right)$ in red, $I_{2}\left(t\right)$ in green, $R_{1}\left(t\right)$ in yellow, $R_{2}\left(t\right)$ in cyan, $R_{3}\left(t\right)$ in black, and $V\left(t\right)$ in brown

In case 1 (v=0.00341 which is equivalent to the actual case in Okinawa so far in which inoculation of 5064 individuals per day is conducted.^2^), as shown in Figure 1A, the infection remains unchanged for some time. However, during that time, antibody carriers $V\left(t\right)$ lose antibodies and its number decreases, and as they are transferred to the susceptible population, $S\left(t\right)$ increases. As $S\left(t\right)$ increases, the number of effective reproductions also increases and exceeds unity, the infection spreads, and the sixth wave arrives. After that, the infected population begins to decrease and the infection changes from spreading to shrinking. However, since $v<d_{1}+d_{2}$, $V\left(t\right)$ gradually loses their antibody and is transferred to $S\left(t\right)$, and then the infection spreads again until it finally converges.

In case 2 (v=0.02 which is equivalent to the case in which inoculation of 29,704 individuals per day is conducted.) which is about 6 times of the number of inoculations in case 1 and satisfies $v>d_{1}+d_{2}$, as shown in Figure 1B, $V\left(t\right)$ increases rapidly from the beginning, and $S\left(t\right)$ quickly settles down. The effective reproduction number remains less than unity and the infection ends without spreading. It can be said that this is a concrete plan of the vaccination policy currently required in Okinawa.

## 4 CONCLUDING REMARKS

It is clear from the above evaluation that vaccination is a key factor in saving lives. In pandemics, it is important to set a vaccination policy that can save lives and maintain daily life at the same time. However, it has reached a difficult stage to deal with infectious diseases by vaccination alone. In the first place, the causes of infectious diseases are environmental problems such as overuse of wild animals, global warming, and deforestation. In order to prevent the outbreak and spread of infectious diseases, it is required to build a sustainable social system based on harmony with the natural environment.

## CONFLICT OF INTEREST

The authors have stated explicitly that there are no conflicts of interest in connection with this article.

## AUTHOR CONTRIBUTIONS

All authors had access to the data and a role in writing the manuscript.
